# Inflammatory markers and pulmonary function in adolescents and young adults 6 months after mild COVID-19

**DOI:** 10.3389/fimmu.2022.1081718

**Published:** 2023-01-06

**Authors:** Silke Lauren Sommen, Lise Beier Havdal, Joel Selvakumar, Gunnar Einvik, Truls Michael Leegaard, Fridtjof Lund-Johansen, Annika E. Michelsen, Tom E. Mollnes, Tonje Stiansen-Sonerud, Trygve Tjade, Vegard Bruun Bratholm Wyller, Lise Lund Berven

**Affiliations:** ^1^ Department of Pediatrics, Akershus University Hospital, Lørenskog, Norway; ^2^ University of Oslo, Oslo, Norway; ^3^ Institute of Clinical Medicine, Faculty of Medicine, University of Oslo, Oslo, Norway; ^4^ Department of Pulmonary Medicine, Akershus University Hospital, Lørenskog, Norway; ^5^ Department of Microbiology and Infection Control, Akershus University Hospital, Lørenskog, Norway; ^6^ Department of Immunology, Oslo University Hospital, Oslo, Norway; ^7^ Research Institute of Internal Medicine, Oslo University Hospital, Oslo, Norway; ^8^ Research Laboratory, Nordland Hospital, Bodø, Norway; ^9^ Centre of Molecular Inflammation Research, Faculty of Medicine and Health Sciences, Norwegian University of Science and Technology, Trondheim, Sør-Trøndelag, Norway; ^10^ Department of Clinical Molecular Biology (EpiGen), University of Oslo and Akershus University Hospital, Lørenskog, Norway; ^11^ Fürst Medical Laboratory, Oslo, Norway

**Keywords:** COVID-19, Post-COVID-19, Long Covid, adolescent, immunology, biomarker, cytokine, pulmonary function

## Abstract

**Introduction:**

Both public and scientific attention have shifted from the acute COVID-19 illness to the chronic disability experienced by a proportion of COVID-19 convalescents. Post COVID-19 condition, a term used for long-lasting symptoms after COVID-19, can affect individuals across all disease severity and age groups. Data on post-COVID-19 symptomatology, epidemiology and pathophysiology in adolescents and young adults are scarce. To date, little is known on the immunological and pulmonary trends in these patients after COVID-19. This study investigated immunological markers and pulmonary function in non-hospitalized patients in this group at 6 months after initial mild COVID-19 infection.

**Methods:**

Non-hospitalized SARS-CoV-2 positive (n = 405) and SARS-CoV-2 negative (n = 111) adolescents and young adults (aged 12-25 years) were followed prospectively for six months after SARS-CoV-2 PCR testing. At baseline and at six months follow-up, all participants underwent an assessment including clinical examination, questionnaires, spirometry, and blood sampling. Cross-sectional comparisons of blood biomarkers; including white blood cell counts, CRP, GDF-15, a 27-multiplex cytokine assay, complement activation products and SARS-CoV-2 antibodies; and spirometry measures were performed after classification of all participants according to their COVID-19 status and adherence to post-COVID-19 case criteria. Associations between biomarkers and COVID-19 symptoms were explored.

**Results:**

No difference in pulmonary function was detected between the groups. COVID-19 convalescents had higher levels of chemokines eotaxin, MCP-1 and IP-10 than non-infected controls. The increase was modest and not associated with long-lasting COVID-19 symptoms.

**Discussion:**

Elevated inflammatory mediators were found in adolescents and young adults six months after mild COVID-19, but there was no association with post-COVID-19 condition.

## Introduction

The clinical outcome of COVID-19, a viral disease caused by SARS-CoV-2, is highly variable. While most affected individuals return to their baseline health status within weeks after the acute respiratory infection, a substantial proportion of convalescents suffers from persisting post-infective symptoms (1–3). Even children and young adults with predominantly mild COVID-19 infection suffer from prolonged symptoms (4). Commonly reported ailments include fatigue, post-exertional malaise, headache, dyspnea, chest tightness and memory problems (4–6). In the literature, these long-lasting sequelae are referred to as Long COVID, Post-Acute Sequelae of COVID-19 (PASC) or Post-COVID-19 condition (PCC), an umbrella term recently defined by the World Health Organization (WHO) as “the condition that occurs in individuals with a history of probable or confirmed SARS-CoV-2 infection, usually 3 months from the onset of COVID-19, with symptoms that last for at least 2 months and cannot be explained by an alternative diagnosis” (7).

The prevalence of long-lasting COVID-19 related symptoms in young people is disputed, with reports indicating fatigue in a range from 0.52% to 66.5% at 3 months post infection in community managed cases (4, 8–10). In addition to the uncertainty of the true prevalence of persistent post-infective symptoms, several questions regarding the pathophysiology and the natural course of the disease remain unanswered. Similarities may be found with other post-infective fatigue syndromes (PIFS) whose clinical presentations overlap greatly with PCC (11). Sustained low-grade inflammation and immune cell activation have been reported in post-infective fatigue after Epstein-Barr virus infection (12, 13). Similarly, investigations into post-COVID-19 immunology in adults find an immune signature with high plasma cytokine levels and altered immune cell populations in convalescent individuals with persistent symptoms compared to immune profiles in recovered convalescents and unexposed controls (14, 15). However, findings between studies vary due to differences in sample size, lack of appropriate control groups, heterogeneous disease definitions and a multitude of laboratory techniques (16). Evidence in young people is scarce, although pro-inflammatory changes with elevated levels of IL-6 and IL-1β have also been shown in PCC affected children (17). Additionally, considering SARS-CoV-2 primarily infects the respiratory tract, respiratory outcomes are relevant. Persisting dyspnea is common in elderly, while prospective data on lung function and respiratory symptoms after COVID-19 in adolescents and young adults are lacking.

Our aim was to investigate immunological blood markers and pulmonary function tests in a large prospective cohort of adolescents and young adults with mild COVID-19 as well as unexposed controls. Thus, we used cross-sectional data from the six-month follow-up appointment and compared markers for the four following groups: affected COVID-19 positive (COVID+ PCC+), recovered COVID-19 positive (COVID+ PCC-), affected COVID-19 negative (COVID- PCC+) and healthy controls (COVID- PCC-). We elucidated whether immunological aberrations compatible with chronic inflammation could be found in PCC affected participants. Furthermore, we investigated associations between inflammatory aberrations and clinical symptom severity.

## Materials and methods

### Study design

LoTECA (Long-term effects of COVID-19 in Adolescents) is a longitudinal, observational cohort study following SARS-CoV-2 positive and negative, non-hospitalized adolescents and young adults over a period of 12 months (ClinicalTrials ID: NCT04686734). Data collection was concluded in June 2022. Details on the study design and baseline findings have been reported elsewhere (18–20). This study reports results from the six months follow-up. The project has been approved by the Norwegian National Committee for Ethics in Medical research (ref. #203645).

### Participants

Individuals aged 12-25 years were recruited consecutively after undergoing a SARS-CoV-2 reverse-transcription polymerase chain reaction (RT-PCR) test at one of two accredited microbiological laboratories (Fürst Medical Laboratory; Dept. of Microbiology and Infection Control, Akershus University Hospital, Oslo, Norway) between December 2020 and May 2021. Exclusion criteria were a) more than 28 days since symptom onset or positive PCR test b) hospitalization due to COVID-19 or c) pregnancy. A total of 405 SARS-CoV-2 positive and 111 SARS-CoV-2 negative individuals were enrolled in the study at baseline. All had undergone testing because of acute infectious symptoms or close contact with SARS-CoV-2-positive cases. Written informed consent was obtained from all participants and/or their legal guardians, in compliance with the Norwegian Health Research Act (ACT 2008-06-20 no. 44).

### Investigational program

At baseline and at six months after recruitment, the study participants were invited for a standardized, one-day investigational program at our study center. All participants underwent a clinical examination and interview, functional testing, completion of questionnaires, and sampling of biological material.

#### Laboratory assays

Blood samples were collected through antecubital venipuncture. Routine blood analyses were carried out by the accredited laboratory at Akershus University Hospital. Blood collected in Vacuette® EDTA (Greiner Bio-One GmbH, Kremsmünster, Austria) tubes was placed in ice-water for 5-60 minutes, centrifuged (10 minutes at 2200 x g) and plasma was aliquoted for storage at -80°C. Blood collected in Vacuette® Serum Separator Clot Activator (Greiner Bio-One GmbH) tubes was left to clot for at least 30 minutes before centrifugation (10 minutes at 2200 x g) and serum aliquotation for storage at -80°C. Plasma samples were assayed on a Multiplex Analyzer (Bio-Rad Laboratories) using a multiplex cytokine assay (Bio-Plex Human Cytokine 27-Plex Panel; Bio-Rad Laboratories Inc., Hercules, CA) containing the following cytokines: IL-1β, IL-1 receptor antagonist, IL-2, IL-4, IL-5, IL-6, IL-7, IL-8, IL-9, IL-10, IL-12, IL-13, IL-15, IL-17A, eotaxin, basic fibroblast growth factor (bFGF), granulocyte colony stimulation factor, granulocyte macrophage colony stimulating factor, interferon-γ, interferon inducible protein (IP-10), monocyte chemotactic protein (MCP-1), macrophage inflammatory protein MIP-1α and MIP-1β, platelet derived growth factor-BB (PDGF-BB), regulated upon activation T cell expressed and secreted (RANTES), tumor necrosis factor (TNF) and vascular endothelial growth factor (VEGF). Plasma levels of growth/differentiation factor 15 (GDF-15) and C-reactive protein (using the high-sensitive technology, hs-CRP) were measured by enzyme immunoassays (EIA) using commercially available antibodies (R&D systems, Minneapolis, MN). Complement activation products, C3bc and the terminal complement complex (TCC) sC5b-9, were quantified in plasma using an in-house enzyme-linked immunosorbent assays (ELISA) based on monoclonal antibodies designed against neoepitopes of the products (21). Epstein-Barr virus (EBV) antibody titers were determined in serum using EBV VCA IgM and IgG and EBV EBNA IgG (LIAISON^®^, DiaSorin, Saluggia, Italy). Samples with inconclusive results from the tests were additionally screened with a rapid chromatographic immunoassay for the qualitative detection of Infectious Mononucleosis heterophile antibodies (Clearview^®^ IM II, Abbott Laboratories, IL). An in-house multiplexed bead-based assay was used to measure SARS-CoV-2 antibodies, including antibodies to the full-length spike protein (Spike-FL), to the receptor-binding domain (RBD) and neutralizing antibodies (by assessment of the effect of sera on the ACE2-binding to RBD) (22).

#### Questionnaires

An extensive questionnaire was used to chart demographic variables, clinical symptoms, personality traits and social factors. The present paper reports results from the Chalder Fatigue Questionnaire (23), the DePaul Symptom Questionnaire (24), and a modified COVID-19 symptom inventory (25, 26). The Chalder Fatigue Questionnaire (CFQ) is a validated tool for the assessment of mental and physical fatigue (27). The 11 CFQ items are rated on a four-point Likert scale (score 0-3) and the CFQ sum score (range 0 to 33) is used here. Scores for the five items addressing post-exertional malaise in the DePaul Symptom Questionnaire were recorded on a five-point Likert scale (score 0 to 4), averaged and multiplied by 25 to generate a total score ranging from 0-100. All items in the COVID-19 symptom inventory were scored on a five-point Likert scale (score 1 to 5) representing symptom frequency (ranging from “never/rarely” to “present all the time”). The sum score for “fever”, “sore throat”, “headache”, “muscle ache” and “fatigue after exercise” represents “infectious symptoms” (total range 5 to 25); whereas “breathlessness”, “cough” and “runny nose” form “respiratory symptoms” (total range 5 to 15); and “memory difficulty”, “concentration difficulty”, “difficulty making decisions” and “confusion/disorientation” make up “cognitive symptoms” (total range 4 to 20).

#### Case assessment

Operationalized case definitions based on the WHO diagnostic definition of PCC (7) and the international criteria for the diagnosis of PIFS (28) were applied to classify all participants as cases or non-cases, regardless of COVID-19 exposure (20). Clinical findings, laboratory reports and questionnaire data from baseline and six months follow-up were scrutinized to identify PCC and PIFS cases. Participants were assigned to one of the four following groups: COVID-19 positive adhering to post-COVID-19 condition criteria (COVID+PCC+), COVID-19 positive not adhering to post-COVID-19 condition criteria (COVID+PCC-), COVID-19 negative adhering to post-COVID-19 condition criteria (COVID-PCC+), and COVID-19 negative not adhering to post-COVID-19 condition criteria (COVID-PCC-). An analogous classification was performed for adherence to PIFS criteria (PIFS+ or PIFS-).

#### Spirometry

Spirometry to measure the forced vital capacity (FVC) and the forced expiratory volume in one second (FEV_1_) was conducted using EasyOne^®^ Air spirometer and EasyOne^®^ Connect software (NDD Medizintechnik AG, Switzerland). Procedures were executed according to the American Thoracic Society and European Respiratory Society guidelines, and individual spirometry recordings that did not satisfy the technical quality requirements were excluded from the analysis (29). The ratio of FEV_1_/FVC was calculated and The Global Lung Function Initiative 2012 network reference values were used to determine the percentage of predicted values and the lower limit of normal (LLN) (30).

### Statistical analysis

In the primary analyses, immunological biomarkers, Epstein-Barr virus and SARS-CoV-2 virus antibodies, and spirometry measures were compared between COVID+PCC+/PIFS+, COVID+PCC-/PIFS-, COVID-PCC+/PIFS+ and COVID-PCC-/PIFS- using Chi square, one-way ANOVA or Kruskal-Wallis tests as appropriate. *Post-hoc* analyses were carried out for comparisons with statistically significant differences between groups. Next, associations between immunological markers and COVID-19 associated symptoms were explored in COVID+PCC+/PIFS+ by the non-parametric Spearman’s rho test. Additionally, all tests were repeated for the COVID-19 positive and negative groups, regardless of PCC/PIFS caseness. A multiple linear regression model was constructed to account for potential confounders in the association between COVID-19 status and biomarker levels.

A total of 20 cytokines (IL-1β, IL-1 RA, IL-2, IL-4, IL-5, IL-6, IL-7, IL-8, IL-10, IL-12, IL-13, IL-15, IL-17A, MIP-1α, bFGF, G-CSF, GM-CSF, IFNγ, PDGF-BB, VEGF) had either a large amount of missing data (> 50%) or detectable but very low values and were removed from the analysis. For the remaining cytokines, values under the lower limit of detection (LDL) were replaced by random values between zero and the LDL for each respective cytokine. TNF had the highest degree of missing data with 32% of values under LDL. Otherwise, no missing data were imputed and per protocol/complete case analysis was carried out after exclusion of a) participants excluded at baseline (n = 7), b) COVID-19 negative individuals at baseline with reported COVID-19 infection in the observational period or SARS-CoV-2 antibodies (any type for unvaccinated, anti-nucleocapsid for vaccinated) detected at six months follow-up or increased anti-nucleocapsid antibody-titer at six months as compared to baseline (n = 16), c) individuals that did not complete the investigational program at six months follow-up (n = 26), d) individuals that had a serological pattern suggesting recently contracted EBV at enrolment or during the observational period (n = 11) and e) individuals that did not provide a blood sample (n = 8).

All variables are reported with mean and standard deviation or median and interquartile range, with corresponding confidence intervals, depending on the distribution. The level of significance was set at 0.05 for two-sided tests and correction for multiple testing was performed. All statistical analysis was carried out in STATA SE version 17.

## Results

At baseline, 516 participants (405 SARS-CoV-2 positive, 111 SARS-CoV-2 negative) consented to participation. At six months follow-up, 448 participants (367 SARS-CoV-2 positive, 81 SARS-CoV-2 negative) met the eligibility criteria and were carried over for further analysis. The participants were evaluated at a median of 212 days (range 179 to 341) after acute COVID-19 symptom onset. An overview of the classification and participant demographics are provided in **Table 1**.

**Table 1:** Cohort characteristics.

Characteristics	COVID19+	COVID19-
Number of samples	367	81
Sex – no. of males (%)	145 (39)	30 (37)
Age at baseline, years – median (IQR)	18 (6)	18 (5)
BMI, kg/m^2^ – mean (SD)	23(4)	23 (4)
Days since symptom onset/positive test – median (range)	213 (162)	210 (83)
PCC case – no. of cases (%)	180 (49)	38 (47)
PIFS case – no. of cases (%)	53 (14)	7 (9)
COVID-19 vaccine° – no. (%)	269 (73)	75 (93)
Tympanic temperature, °C – mean (SD)	36.6 (0.38)	36.7 (0.36)
Sp02, % - median (IQR)	99 (1)	98 (2)
Respiratory frequency, breaths/min – median (IQR)	16 (6)	14 (6)
FVC, L - mean (SD)	4.3 (1.0)	4.4 (0.80)
FEV_1_, L - mean (SD)	3.7 (0.8)	3.7 (0.6)
Blood haemoglobin, g/dL – mean (SD)	13.6 (1.2)	13.7 (1.0)
Serum CRP*, mg/L, no. (%) <1 1-5 >5	257 (70) 91 (25) 19 (5)	44 (54) 30 (37) 7 (9)
Plasma ferritin, μg/L – median (IQR)	44 (46)	44 (30)
Plasma IgA, g/L – mean (SD)	1.8 (0.77)	1.7 (0.75)
Plasma IgG, g/L – mean (SD)	11 (2.1)	11 (2.0)
Plasma IgM, g/L – mean (SD)	1.3 (0.50)	1.2 (0.51)
Plasma TSH, mIE/L – mean (SD)	1.9 (0.9)	1.8 (1.0)
Plasma T4, pmol/L	16 (2.1)	16 (1.9)
Plasma ALT, U/L – mean (SD)	19 (9.1)	17.9 (6.8)
Serum potassium, mmol/L – mean (SD)	4.0 (0.23)	4.1 (0.28)
Serum sodium, mmol/L – mean (SD)	139 (1.8)	139 (1.8)
Plasma creatinine, μmol/L – mean (SD)	68 (13)	68 (12)
Plasma CK, U/L – median (IQR)	114 (108)	114 (122)
Venous blood pH – mean (SD)	7.4 (0.03)	7.4 (0.03)
Venous blood $\text{HC}{\text{O}}_{3}^{-}$ , mmol/L – mean (SD)	26 (2.0)	25 (2.2)
Venous blood PvCO_2_, kPa – median (IQR)	6.4 (1.2)	6.2 (1.4)

IQR, interquartile range; SD, standard deviation; BMI, body mass index; PCC, post-COVID-19 condition; PIFS, post-infective fatigue syndrome; °received at least one COVID-19 vaccine dose; FVC, forced vital capacity; FEV_1_, forced expiratory volume 1 second; *Serum CRP levels are below the lower limit of detection (<0.6) in many of the participants and are therefore reported as frequencies within categories, maximum observation of 118 mg/L in COVID-19 positive group.

### Comparison of post-COVID-19 condition cases and non-cases

**Figure 1:**
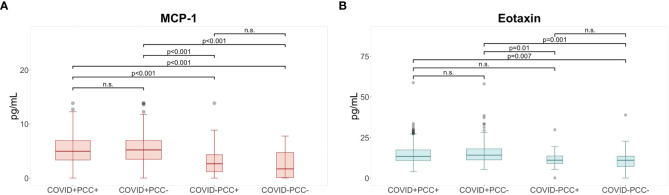
Elevated levels of cytokines at six months following COVID-19. Post hoc analysis for cytokines with significant differences between post COVID-19 condition (PCC) groups shows **(A)** higher levels of MCP-1° and **(B)** higher levels of eotaxin* in the COVID+ PCC+ and COVID+ PCC- groups compared to participants in the COVID- PCC+ and COVID- PCC- groups at 6 months. °Based upon pairwise comparison of means with Bonferroni adjustment for test multiplicity; *based on Dunn**’**s test for pairwise comparisons with Benjamini-Hochberg adjustment for test multiplicity; p-values indicate comparisons rejected by these methods at the alpha level (two sided tests); MCP, monocyte chemotactic protein; n.s., non-significant.

Background characteristics were similar across the two groups, including the adherence to WHO Post-COVID-19 condition criteria, with 49.0% in the COVID-19 positive and 46.9% in the COVID-19 negative group (**Table 1**). Only COVID vaccine uptake was significantly higher in the COVID-19 negative group compared to the COVID-19 positive group (Chi Square test χ^2 =^ 13.86, p-value <0.001, **Table 1**). The COVID+PCC+ and COVID+PCC- participants had significantly higher plasma levels of eotaxin and MCP-1 compared to COVID- PCC- (**Table 2**; **Figure 1**). Fold increases were 2.9 for MCP-1 and 1.3 for eotaxin respectively, when comparing COVID+PCC+ to COVID-PCC-. Additionally, COVID+PCC+ participants showed elevated levels of MCP-1 compared to COVID-PCC+, who in turn had significantly higher levels than COVID-PCC- (**Table 2**; **Figure 1**). There were no statistically significant differences in other immunological biomarkers, nor in the differential white blood cell counts across the four groups (**Table 2**). The comparison of spirometry measures did not show any differences (**Table 3**). The COVID vaccine uptake in the COVID-19 positive group was similar for those who developed PCC compared to those who did not (**Table 4**). Among participants with prior infection, there were no differences in SARS-CoV-2 antibody titers between unvaccinated PCC+ and PCC-, nor were there any differences between vaccinated PCC+ and vaccinated PCC- (**Table 5**). SARS-CoV-2 nucleocapsid antibody levels declined between the baseline visit and the 6 months follow-up in both the COVID+PCC+ and COVID+PCC- groups (**Supplementary Figure 1**; **Supplementary Table 1**). Furthermore, there was no difference in overall Epstein-Barr virus seroprevalence in the four PCC groups (**Supplementary Table 2**).

**Table 2:** Cross-sectional analysis of white blood cell counts, cytokines and complement activation markers between post COVID-19 (PCC) groups.

	LDL (pg/mL)	COVID+ PCC+ (n=180)	COVID+ PCC-(n=187)	COVID- PCC+ (n=38)	COVID- PCC-(n=43)	p-value
Plasma TNF, pg/mL – median (IQR) C.I	3.0	6.9 (14) 5.6 to 9.4	9.4 (15.3) 6.9 to 10.7	8.2 (14.2) 0.44 to 10	0.49 (14) 0.32 to 7.4	0.07*
Plasma MCP-1, pg/mL – median (IQR) C.I.	0.40	5.0 (3.6) 4.4 to 5.6	5.2 (3.5) 4.9 to 5.7	2.7 (3.4) 1.8 to 3.5	1.7 (4.9) 1.1 to 3.0	0.0001*
Plasma IP-10, pg/mL – median (IQR) C.I.	1.0	106 (64) 100 to 114	104 (68) 97 to 116	97 (49) 82 to 112	90 (62) 72 to 105	0.10*
Plasma Eotaxin, pg/mL – mean (SD) C.I.	0.30	15 (6.7) 14 to 16	16 (6.6) 15 to 16	11 (5.0) 10 to 13	11 (6.6) 9.2 to 13.2	**0.0001°**
Plasma MIP-1β, pg/mL – median (IQR) C.I.	0.30	28 (35) 24 to 34	27 (37) 24 to 31	25 (38) 15 to 34	23 (25) 16 to 30	0.22*
Plasma RANTES, pg/mL – median (IQR) C.I.	3.0	141 (174) 124 to 162	139 (154) 127 to 152	100 (198) 90 to 182	129 (132) 88 to 147	0.28*
Plasma IL-9, pg/mL – median (IQR) C.I.	2.0	100 (153) 83 to 125	96 (150) 82 to 110	74 (179) 54 to 151	75 (86) 51 to 97	0.24*
Plasma GDF-15, ng/mL mean (SD) C.I.		0.45 (0.14) 0.44 to 0.47	0.44 (0.11) 0.42 to 0.46	0.44 (0.11) 0.40 to 0.47	0.44 (0.12) 0.40 to 0.47	0.61°
Plasma hs-CRP, μg/mL – median (IQR) C.I.		1.3 (5.3) 0.98 to 1.82	1.2 (3.0) 0.83 to 1.5	1.8 (4.6) 0.83 to 3.1	2.7 (5.6) 0.95 to 4.6	0.20*
Plasma C3bc, ng/mL – mean (SD) C.I.		3.9 (1.3) 3.7 to 4.1	3.9 (1.6) 3.6 to 4.1	3.3 (1.2) 2.9 to 3.7	3.8 (1.7) 3.2 to 4.3	0.19°
Plasma TCC, CAU/mL – median (IQR) C.I.		0.2 (0.2) 0.2 to 0.2	0.19 (0.13) 0.18 to 0.20	0.2 (0.15) 0.15 to 0.24	0.18 (0.21) 0.14 to 0.26	0.99*
Blood Leukocyte count, 10^9^ cells/L - mean (SD) C.I.		6.3 (1.8) 6.1 to 6.6	5.9 (1.7) 5.6 to 6.1	5.9 (1.7) 5.3 to 6.5	5.9 (1.36) 5.4 to 6.3	0.08°
Blood Lymphocyte count, 10^9^ cells/L - mean (SD) C.I.		2.0 (0.5) 1.9 to 2.1	2.0 (0.58) 1.9 to 2.1	1.9 (0.44) 1.8 to 2.1	2.0 (0.45) 1.9 to 2.1	0.90°
Blood Monocyte count, 10^9^ cells/L - mean (SD) C.I.		0.50 (0.16) 0.47 to 0.51	0.5 (0.1) 0.4 to 0.5	0.46 (0.13) 0.42 to 0.51	0.45 (0.13) 0.41 to 0.50	0.30°
Blood Neutrophil count, 10^9^ cells/L – mean (SD) C.I.		3.7 (1.6) 3.4 to 3.9	3.2 (1.4) 3.0 to 3.4	3.3 (1.4) 2.8 to 3.8	3.2 (1.14) 2.9 to 3.6	0.03°
Blood Eosinophil count, 10^9^ cells/L - mean (SD) C.I.		0.19 (0.14) 0.17 to 0.21	0.19 (0.22) 0.16 to 0.22	0.20 (0.16) 0.14 to 0.25	0.19 (0.16) 0.14 to 0.24	0.99°
Blood Platelets count, 10^9^ cells/L - mean (SD) C.I.		276 (59) 267 to 285	264 (58) 255 to 273	288 (65) 267 to 310	266 (50) 250 to 281	0.06°
Neutrophil-to-Lymphocyte ratio – mean (SD) C.I.		1.94 (1.06) 1.79 to 2.10	1.7 (0.88) 1.6 to 1.8	1.8 (0.72) 1.5 to 2.0	1.7 (0.67) 1.5 to 1.9	0.07°
SII - median (IQR) C.I.		445 (318) 411 to 475	382 (261) 351 to 415	451 (345) 361 to 562	386 (315) 321 to 488	0.0095*

Based upon Chi Square test; *Based upon Kruskal-Wallis one way analysis; °based upon one way ANOVA analysis; LDL, lower detection limit for multiplex assay; IQR, interquartile range; SD, standard deviation; GDF, growth/differentiation factor; IL, interleukin; TNF, tumor necrosis factor; MCP, monocyte chemotactic protein; IP, interferon gamma-induced protein; MIP, macrophage inflammatory protein; hsCRP, high-sensitive assay of C-reactive protein; RANTES, Regulated on activation, normal T-cell expressed and secreted; C3b, complement component 3 part bc; TCC, terminal complement complex; CAU, complement activation unit; SII, Systemic inflammatory index = Neutrophils x Platelets/Lymphocytes; Statistically significant p-values after application of Benjamini Hochberg correction (FDR 0,05) for multiple testing are highlighted in **bold**.

**Table 3:** Cross-sectional comparison of spirometry results between post COVID-19 (PCC) groups.

	COVID+ PCC+	COVID+ PCC-	COVID- PCC+	COVID- PCC-	p-value
Number of samples	132	144	32	31	
FVC, % of predicted - mean (SD)C.I.	99 (11) 98 to 101	101 (13) 99 to 103	104 (9.2) 100 to 107	101 (9.54) 97 to 104	0.26°
FVC < LLN - no. (%)C.I.	10 (7.7) 4.2 to 14	5 (3.6) 1.5 to 8.3	0 (0) n.a.	1 (3.5) 0.48 to 21	0.22*
FEV_1_, % of predicted - mean (SD)C.I.	97 (11) 95 to 99	99 (12) 97 to 101	102 (7.3) 99 to 105	97 (9.1) 94 to 101	0.14°
FEV_1_-to-FVC ratio - mean (SD)C.I.	0.85 (0.07) 0.84 to 0.86	0.85 (0.06) 0.84 to 0.86	0.86 (0.05) 0.84 to 0.88	0.84 (0.06) 0.82 to 0.86	0.82°
FEV_1_-to-FVC ratio < 0.7 - no. (%)C.I.	4.0 (3.1) 1.2 to 7.9	2.0 (1.4) 0.35 to 5.5	1.0 (3.3) 0.46 to 20	0 (0) n.a.	0.63*

Based upon one way ANOVA analysis; *Based upon Chi-square test; SD, standard deviation; FVC, forced vital capacity; LLN, lower limit of normal; FEV_1_, forced expiratory volume 1 second; n.a., non-applicable.

**Table 4:** Vaccination and PCC status in COVID-19 positive.

Vaccination	PCC+ (%)	PCC- (%)
**Yes (%)**	136 (37)	133 (36)
**No (%)**	44 (12)	54 (15)

Vaccination is determined as at least one COVID-19 vaccine dose. % = percentage of total sample. Chi square test p=0,35.

Table 5Cross-sectional comparison of SARS-CoV-2 antibodies between COVID-19 positive PCC groups.

**Table:** 

	*unvaccinated PCC+*	*Vs.*	*unvaccinated PCC-*	*p-value*
BAU/mL – median (IQR)	144 (1278)		121 (1210)	0.48
Nucleocapsid Antibody – median (IQR)	14 (21)		15 (28)	0.63

**Table:** 

	*vaccinated PCC+*	*Vs.*	*vaccinated PCC-*	*p-value*
BAU/mL – median (IQR)	7436 (11295)		7985 (13958)	0.56
Nucleocapsid Antibody – median (IQR)	7.1 (12)		6.4 (8.9)	0.35

Based upon Mann Whitney; BAU, Binding Antibody Units; IQR, interquartile range

**Figure 2:**
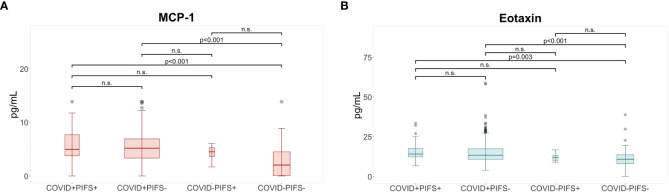
Elevated levels of cytokines at six months following COVID-19. *Post hoc* analysis for cytokines with significant differences between post-infective fatigue syndrome (PIFS) groups shows **(A)** higher levels of MCP-1° and **(B)** higher levels of eotaxin* in the COVID+ PIFS+ and COVID+ PIFS- groups compared to participants in the COVID- PIFS+ and COVID- PIFS- groups at 6 months. ° Based upon pairwise comparison of means with Bonferroni adjustment for test multiplicity; * based on Dunn’s test for pairwise comparisons with Benjamini-Hochberg adjustment for test multiplicity; p-values indicate comparisons rejected by these methods at the alpha level (two sided tests); MCP, monocyte chemotactic protein; n.s., non-significant.

Analogously, when classified according to PIFS criteria, levels of MCP-1 and eotaxin were elevated in the COVID+PIFS+ and COVID+PIFS- versus COVID-PIFS- (**Supplementary Table 3**; **Figure 2**). Fold changes were 2.4 for MCP-1 and 1.36 for eotaxin respectively when comparing COVID+PIFS+ participants to COVID-PIFS-. Comparison of peripheral white blood cell counts, spirometry measures and SARS-CoV-2 antibodies did not reveal any significant differences between the four groups (**Supplementary Tables 3**–**5**).

### Associations within the post COVID-19 condition cases

Within the COVID+PCC+ group, there was no correlation between COVID-19 associated symptoms and MCP-1 or eotaxin levels (**Table 6**). Analogously, there were no significant associations between these markers and COVID-19 associated symptoms in the affected COVID-19 positive if classified according to PIFS criteria (COVID+PIFS+) (**Supplementary Table 6**). When looking at PCC caseness regardless of COVID-19 status, all correlations were non-significant (**Supplementary Table 7**).

**Table 6:** Correlation between immunological markers and symptoms within the COVID+ PCC+ group.

COVID-19 associated symptoms						
		*Airway symptom score^a^*	*Infection symptom score^b^*	*Cognitive symptom score^c^*	*Post-exertional malaise score^d^*	*Fatigue score^e^*
MCP-1	Corr. coef. (rho) p-value	0.0024 0.9750	0.0098 0.8965	-0.0024 0.9748	0.0936 0.2113	0.0498 0.5071
Eotaxin Corr. coef. (rho) p-value		0.0881 0.2398	0.1343 0.0723	0.0760 0.3105	0.0899 0.2303	0.1180 0.1147

Spearman’s rho correlation analysis with significant p-values marked in bold after Benjamini Hochberg correction (FDR 0,05) for test multiplicity. ^a^ Sum of scores across the items “breathlessness”, “cough” and “runny nose”. ^b^ Sum of scores across the items “fever/chills”, “sore throat”, “headache”, “muscle ache” and “fatigue after exercise”. ^c^ Sum of scores across the items “decision-making”, “memory problems”, “concentration difficulty” and “confusion/disorientation”. ^d^ Sum of the DePaul Symptom Questionnaire items relating to post-exertional malaise. ^e^ Numerical sum of scores on the Chalder Fatigue questionnaire.

### Comparison of COVID-19 positive and COVID-19 negative participants

**Figure 3:**
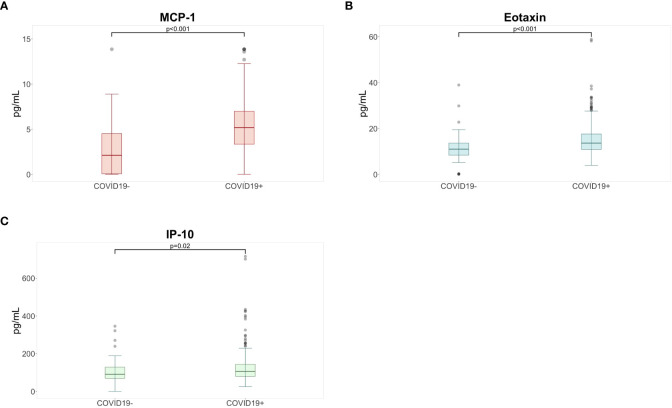
Higher levels of **(A)** MCP-1°, **(B)** eotaxin*, and **(C)** IP-10° were detected in the COVID-19 positive group compared to participants in the COVID-19 negative group at six months following COVID-19 infection. ° based upon Mann Whitney U with Benjamini Hochberg correction for multiple testing; * based upon Student T test with Benjamini Hochberg correction for multiple testing; p-values indicate comparisons rejected by these methods at the alpha level (two sided tests); IP, interferon gamma induced protein; MCP, monocyte chemotactic protein.

The COVID-19 positive group had significantly higher levels of plasma MCP-1, eotaxin and IP-10 compared to the COVID-19 negative group (**Supplementary Table 8**; **Figure 3**). Fold changes were 2.47, 1.31 and 1.16 for MCP-1, eotaxin and IP-10 respectively. To confirm whether COVID-19 status was significantly associated with these plasma levels after accounting for demographic differences, a multiple linear regression was performed incorporating age, sex, body-mass-index, and COVID-19 vaccination status (**Supplementary Table 9**). In addition, the frequency of asthmatics was similar in both COVID-19 groups (Chi square test, p-value = 0,63). No significant differences between COVID-19 groups were detected when comparing the differential white blood cell count and spirometry measures (**Supplementary Tables 8**, **10**). Within the COVID-19 positive group, none of the immunological markers were correlated with COVID-19 associated symptoms (**Supplementary Table 11**).

## Discussion

This study of a large group of young, non-hospitalized COVID-19 convalescents shows that a biomarker signature remains at six months after mild COVID-19 infection. Elevated levels of eotaxin, MCP-1 and IP-10 were detected in COVID-19 convalescents compared to COVID-19 negative participants. However, these changes in immunological markers did not associate with persistent symptoms or disability experienced by a subset of study participants after mild COVID-19 infection. No alterations in immunological markers were exclusively detected in post-COVID-19 affected participants, regardless of the applied post-COVID-19 definition. This resonates well with the baseline report from our cohort where clinical symptoms were independent of inflammatory markers (18). Additionally, no differences were detected in spirometry measures between COVID-19 positive and negative participants, regardless of post-COVID-19 symptomatology. Thus, complaints of dyspnea in post-COVID-19 affected individuals could not be explained by deterioration of pulmonary function, nor by an inability to repair tissue damage. This is in line with previously reported findings from the sub-acute infection stage of our cohort (18). To the best of our knowledge, this is the largest prospective study assessing post-COVID-19 immune alterations and pulmonary function in adolescents and young adults.

Different hypotheses have been proposed for PCC pathogenesis, including antigen persistence (31), latent virus reactivation (32), chronic inflammation and auto-immunity (33). Our data suggest an immune signature specific to COVID-19 but unrelated to post-COVID-19 symptomatology. Multiplex plasma cytokine analysis revealed elevated levels of three chemokines (monocyte chemoattractant protein 1 (MCP-1), eotaxin, and interferon-γ induced protein (IP-10)) in COVID-19 positive participants. IP-10 and eotaxin were also elevated in the COVID-19 group during the sub-acute infection stage (18). Chemokines are a superfamily of proteins that mediate a range of pro-inflammatory effects on leukocytes, such as chemotaxis, degranulation, and integrin activation. Eotaxin, an eosinophil-specific chemoattractant belonging to the CC family of chemokines, is implicated in the accumulation and activation of eosinophils at sites of allergic inflammation (34). MCP-1 is structurally closely related to eotaxin, also belonging to the CC family, but principally attracts monocytes, activated T cells and basophils (35). MCP-1, together with IP-10, a CXC family member, has been brought forward as part of the “cytokine storm” in COVID-19, a type of hyperinflammatory state associated with worse outcome in acute SARS-CoV-2 infection (6). Upon viral infection, IP-10 is upregulated by locally produced IFN-γ and becomes available for the recruitment of monocytes, T lymphocytes and NK cells as part of the anti-viral response (35). Taken together, while chemokines are essential in the initial phases of antiviral response, sustained elevated levels can produce hyperinflammation and tissue damage.

One hypothesized explanation for the persistent elevation of these chemokines is viral persistence in COVID-19 convalescents, where the pathogen chronically stimulates the innate and adaptive immune system (31). In our cohort, SARS-CoV-2 anti-nucleocapsid titers declined over time and anti-SARS-CoV-2 specific antibodies were similar between unvaccinated convalescents with or without persistent post-infectious symptoms, decreasing the likelihood of antigen persistence as a trigger of PCC. This contrasts with findings by Klein et al. showing enhanced anti-Spike immune response among PCC+ participants (36). However, conventional antibody screening in peripheral blood might not be sufficient to detect viral persistence in “anatomical sanctuaries”. Previously, SARS-CoV-2 nucleocapsid protein and viral RNA have been detected in gut tissue after COVID-19 recovery (37).

Recent studies have found immunological markers and immune cell populations that remain altered following COVID-19 infection, some changes being specific to PCC. Bergamaschi et al. identified persistent abnormal levels of IL-6 and TNF after clinical recovery from COVID-19 infection (38). Phetsouphanh et al. identified six cytokines (IFN-β, IFN-λ1, IFN-γ, CXCl9, CXCL10, IL-8, sTIM-3) that were elevated in the COVID19+ PCC+ and PCC- versus controls at four months after COVID-19 infection, pointing to an immune signature specific to COVID-19 (14). Interestingly, at eight months IFN-β and IFN-λ levels were significantly elevated in PCC+ compared to all other groups, indicating that PCC related immune alterations might become more apparent with time. These findings align with a recent study assessing PCC immune profiles at one year after infection: marked alterations in immune cell population frequencies are accompanied by higher intracellular IL-4 and IL-6 production in peripheral blood mononuclear cells (PBMC) from PCC+ patients, together with elevated IL-8 and CCL4 plasma levels (36). While the exact immunological markers differ between studies, all indicate an ongoing inflammatory response and immune dysregulation in COVID-19 convalescents weeks-to-months after the initial SARS-CoV-2 infection. Variations in the immune markers on the cellular and transcriptional levels are attributed to persistent symptoms in PCC+ convalescent individuals (33, 36, 39).

In contrast, our data did not reveal immunological aberrations specific to PCC, nor did we find any relationship between immunological markers and symptom severity. Our study is based on a prospective cohort of non-hospitalized adolescents who have been rigorously assessed with operationalized PCC and PIFS criteria. Given the high prevalence of PCC-associated symptoms in the general population (40), our study might have captured PCC cases too liberally and therefore lacked power to detect differences in immune markers in true PCC cases. The broad PCC case definition by the WHO can make it difficult to distinguish between affected individuals due to the impact of the pandemic-associated stress or due to the impact of the infection itself. However, the analysis has been repeated with case assessment based on post-infective fatigue syndrome (PIFS) criteria and the classification was done blinded to COVID-19 status, increasing the validity of our findings. Phetsouphanh et al. have based their PCC classification on the presence of few hallmark symptoms (cough, fatigue, dyspnea) (14), while Klein et al. have recruited their PCC participants from Long COVID clinics (36), increasing the risk that observed differences between PCC, convalescents and controls are due to unrecognized factors (age, co-morbidities, initial disease severity) rather than actual differences in immunological markers. Self-selection, for instance, can lead to inclusion of PCC patients representing the extremes of the symptom spectrum related to post-COVID-19 symptomatology and produce biased findings. All findings, including our own, should be validated in a comparable cohort.

There are several limitations to our study. Our analysis is based on a small number of COVID-19 negative participants, many of which were recruited after SARS-CoV-2 testing for infectious symptoms. They might have contracted a different viral disease and therefore may not be true controls. However, it is assumed that common colds do not provoke post-infectious fatigue states (41) and participants with recent Epstein-Barr virus infection, a known precipitator of post-infective fatigue (42), were excluded from the analysis. In addition, there is a constantly evolving SARS-CoV-2 variant landscape, and B1.1.7. (alpha) was the dominant SARS-CoV-2 virus variant throughout the recruitment period for this study. The impact of the delta and omicron variants cannot be assessed here as the disease burden and pathophysiology might be variant-dependent. Finally, only seven out of 27 cytokines assessed by the multiplexed analysis yielded levels sufficient for conclusive analysis, and only three showed statistically significant differences between the groups. This is not surprising as peripheral blood cytokine levels are often undetectable with standard assays in healthy individuals (43). Reservations should be made regarding (over)interpretation of these results as the differences in cytokine levels were modest and fell within the physiological range for healthy individuals (44). Most participants received a COVID-19 vaccine during the follow-up period, potentially influencing the peripheral immune markers to a greater extent than the mild COVID-19 infection itself. Hence, the biological and clinical relevance of our findings is not clear, especially given the lack of association with post-COVID-19 symptomatology. Cytokine levels in plasma might not reliably reflect those in inflamed tissues and therefore, fail to show an association between tissue damage, inflammation, and Post-COVID-19 symptomatology. Other techniques, ultra-sensitive cytokine assays and single cell transcriptomics might still shed light on immune cell populations or biomarkers associated with PCC.

In the future, more prospective studies are needed to investigate the drivers of immune activation in both SARS-CoV-2 convalescence and Post-COVID-19 condition. The focus should lie on both high-resolution immune profiling and appropriate study design, including comparisons with normative data on post-COVID-19 symptomatology and adequate post-COVID-19 condition case ascertainment.

## Conclusion

SARS-CoV-2 virus exerted a modest, prolonged effect on the expression of chemokines eotaxin, MCP-1 and IP-10 at 6 months after convalescence. These elevated immunological markers were, however, unrelated to post-COVID-19 symptomatology.

## Data availability statement

The raw data supporting the conclusions of this article will be made available by the authors, without undue reservation.

## Ethics statement

The studies involving human participants were reviewed and approved by Regionale Komiteer for Medisinsk og Helsefaglig Forskningsetikk (REK). Written informed consent to participate in this study was provided by the participants’ legal guardian/next of kin.

## Author contributions

The authors confirm contribution to the paper as follows: Study conception and design: VW. Data collection: SS, LB, JS, LH, TS-S. Analysis and interpretation of results: SS, LB, VW, LH, JS, FL-J, TM, AM, GE, TL, TT. Draft manuscript preparation: SS, LB, VW. All authors contributed to the article and approved the submitted version.
