# Supplementary material for: Inflammatory markers and pulmonary function in adolescents and young adults 6 months after mild COVID-19
Source: Front Immunol. 2023 Jan 6;13:1081718. doi: 10.3389/fimmu.2022.1081718 (PMC9853911; doi:10.3389/fimmu.2022.1081718)
Supplement: Supplementary file 1 [file DataSheet_1.pdf]

Supplementary Material

Supplementary Tables

Suppl. Table 1 - Correlation analysis for nucleocapsid titers in COVID19**+**

			Nucleocapsid (baseline)	***Eotaxin***	***MCP-1***	***IP-10***
Nucleocapsid (6m)	vaccinated	Rho	0.53	-0.0963	0.0532	0.1178
		p-value	**<0.001**	0.1185	0.3903	0.0559
	unvaccinated	Rho	0.52	-0.1341	-0.1948	-0.1735
		p-value	**0.0000**	0.1928	0.0571	0.0909
	total	Rho	0.53	-0.1631	0.0140	0.0639
		p-value	**<0.0001**	**0.0019**	0.7905	0.2262

Based upon Spearman's rho correlation analysis with significant p-values after Benjamini-Hochberg correction (FDR 0,05) for multiple testing highlighted in bold.

Suppl. Table 2 - Cross-sectional comparison of Epstein-Barr virus serology between PCC groups.

	COVID+ PCC+	***COVID+ PCC-***	***COVID- PCC+***	***COVID- PCC-***	***p-value***
Positive EBV serology at baseline (%)	130 (73)	143 (77)	23 (61)	27 (63)	0.10**

** Based upon chi square test. Participants with recent Epstein-Barr virus infection in the observational period were excluded from analysis.

Suppl. Table 3 – Cross-sectional comparison of white blood cell counts, cytokines and complement activation products between post-infective fatigue syndrome (PIFS) groups.

	LDL (pg/mL)	*COVID+ PIFS+ (n=53)*	*COVID+ PIFS- (n=314)*	*COVID- PIFS+ (n=7)*	*COVID- PIFS- (n=74)*	*p-value*
Plasma TNF, pg/mL – median (IQR)<br>C.I.	3.0	12 (19)<br>5.6 to 16	8.2 (14)<br>5.7 to 9.4	13 (22)<br>0.97 to 38	4.8 (13)<br>0.36 to 8.2	0.03*
Plasma MCP-1, pg/mL – median (IQR)<br>C.I.	0.40	5.0 (4.0)<br>4.5 to 6.9	5.2 (3.6)<br>4.6 to 5.6	4.5 (2.5)<br>2.3 to 6.1	2.1 (4.4)<br>1.4 to 2.7	**0.0001***
Plasma IP-10, pg/mL – median (IQR)<br>C.I.	1.0	114 (71)<br>106 to 131	103 (64)<br>99 to 108	91 (41)<br>67 to 142	91 (61)<br>81 to 103	0.0740*
Plasma Eotaxin, pg/mL – mean (SD)<br>C.I.	0.30	16 (5.4)<br>14 to 17	15 (6.8)<br>14 to 16	12 (2.7)<br>9.6 to 15	11 (6.1)<br>10 to 13	**0.0001°**
Plasma MIP-1β, pg/mL – median (IQR)<br>C.I.	0.30	38 (59)<br>26 to 44	26.3 (34)<br>24 to 30	34 (57)<br>14 to 126	23 (25)<br>16 to 28	0.0386*
Plasma RANTES, pg/mL – median (IQR)<br>C.I.	3.0	197 (315)<br>129 to 231	137 (148)<br>126 to 148	259 (349)<br>54 to 733	117 (127)<br>94 to 140	0.05*
Plasma IL-9, pg/mL – median (IQR)<br>C.I.	2.0	143 (281)<br>72 to 208	95 (131)<br>84 to 105	162 (330)<br>24 to 633	72 (95)<br>57 to 95	0.08*
Plasma GDF-15, ng/mL mean (SD)<br>C.I.		0.46 (0.13)<br>0.42 to 0.49	0.44 (0.12)<br>0.43 to 0.46	0.41 (0.09)<br>0.33 to 0.49	0.44 (0.12)<br>0.41 to 0.47	0.68°
Plasma hs-CRP, µg/mL – median (IQR)<br>C.I.		1.2 (4.0)<br>0.78 to 2.0	1.2 (3.7)<br>0.99 to 1.6	2.1 (4.9)<br>0.38 to 12	1.99 (5.2)<br>1.02 to 3.6	0.51*
Plasma C3bc, ng/mL – mean (SD)<br>C.I.		3.5 (0.97)<br>3.3 to 3.8	3.9 (1.6)<br>3.8 to 4.1	3.32 (0.70)<br>2.67 to 3.97	3.6 (2.6)<br>3.2 to 4.0	0.09°
Plasma TCC, CAU/mL – median (IQR)<br>C.I.		0.17 (0.13)<br>0.16 to 0.22	0.19 (0.13)<br>0.18 to 0.21	0.17 (0.21)<br>0.10 to 0.45	0.20 (0.18)<br>0.15 to 0.24	0.99*
Blood Leukocyte count, 10^9 cells/L - mean (SD)<br>C.I.		6.5 (1.8)<br>6.0 to 7.0	6.0 (1.8)<br>5.8 to 6.2	5.9 (1.9)<br>3.92 to 7.8	5.9 (1.5)<br>5.5 to 6.2	0.20°
Blood Lymphocyte count, 10^9 cells/L - mean (SD)<br>C.I.		2.0 (0.51)<br>1.9 to 2.2	2.0 (0.55)<br>1.9 to 2.1	1.83 (0.38)<br>1.43 to 2.24	2.0 (0.5)<br>1.9 to 2.1	0.86°
Blood Monocyte count, 10^9 cells/L - mean (SD)<br>C.I.		0.49 (0.16)<br>0.44 to 0.53	0.47 (0.15)<br>0.46 to 0.49	0.48 (0.21)<br>0.26 to 0.71	0.46 (0.13)<br>0.43 to 0.49	0.70°
Blood Neutrophil count, 10^9 cells/L - mean (SD)<br>C.I.		3.8 (1.6)<br>3.4 to 4.3	3.4 (1.5)<br>3.2 to 3.5	3.43 (1.3)<br>2.04 to 4.83	3.2 (1.3)<br>2.9 to 3.5	0.14°
Blood Eosinophil count, 10^9 cells/L – mean (SD)<br>C.I.		0.19 (0.15)<br>0.14 to 0.23	0.19 (0.19)<br>0.17 to 0.21	0.17 (0.08)<br>0.08 to 0.25	0.19 (0.16)<br>0.15 to 0.23	0.98°
Blood Platelets count, 10^9 cells/L - mean (SD)<br>C.I.		282 (71)<br>263 to 302	268 (56)<br>262 to 274	329 (69)<br>256 to 401	271 (56)<br>259 to 285	0.03°
Neutrophil-to-Lymphocyte ratio – mean (SD)<br>C.I.		2.0 (1.0)<br>1.8 to 2.3	1.8 (0.97)<br>1.7 to 1.9	1.93 (0.78)<br>1.11 to 2.74	1.7 (0.69)<br>1.5 to 1.9	0.23°

SII - median (IQR)<br>C.I.	468 (408)<br>393 to 655	408 (264)<br>384 to 432	528 (438)<br>366 to 950	402 (317)<br>358 to 474	0.05*

• Based upon Chi Square test; *Based upon Kruskal-Wallis one way analysis; °based upon one way ANOVA analysis; LDL, lower detection limit for multiplex assay; IQR, interquartile range; SD, standard deviation; GDF, growth/differentiation factor; IL, interleukin; TNF, tumor necrosis factor; MCP, monocyte chemotactic protein; IP, interferon gamma-induced protein; MIP, macrophage inflammatory protein; hsCRP, high-sensitive assay of C-reactive protein; RANTES, Regulated on activation, normal T-cell expressed and secreted; C3b, complement component 3, part bc; TCC, terminal complement complex; CAU, complement activation unit; SII = Neutrophils x Platelets/Lymphocytes; Statistically significant p-values after application of Benjamini Hochberg correction (FDR 0,05) for multiple testing are highlighted in bold.

Suppl. Table 4 – Cross-sectional comparison of spirometry results between PIFS groups.

	COVID+ PIFS+	*COVID+ PIFS-*	*COVID- PIFS+*	*COVID- PIFS-*	*p-value*
Number of samples	35	241	6	57	
FVC, % of predicted - mean (SD)<br>C.I.	99 (9.2)<br>95 to 102	100 (12)<br>99 to 102	104 (4.5)<br>97 to 111	102 (9.7)<br>100 to 105	0.47°
FVC < LLN - no. (%)<br>C.I.	4.0 (11)<br>4.3 to 27	11 (4.7)<br>2.6 to 8.2	0 (0)<br>n.a.	1.0 (1.8)<br>0.25 to 12	0.20*
FEV_1, % of predicted - mean (SD)<br>C.I.	96 (12)<br>92 to 100	98 (12)<br>97 to 100	105 (2.06)<br>102 to 108	99 (8.6)<br>97 to 102	0.39°
FEV_1-to-FVC ratio - mean (SD)<br>C.I.	0.85 (0.08)<br>0.83 to 0.88	0.85 (0.06)<br>0.84 to 0.86	0.88 (0.04)<br>0.83 to 0.94	0.85 (0.06)<br>0.83 to 0.86	0.70°
FEV_1-to-FVC ratio < 0.7 - no. (%)<br>C.I.	1.0 (2.9)<br>0.4 to 0.2	5.0 (2.1)<br>0.88 to 5.0	0 (0)<br>n.a.	1.0 (1.8)<br>0.25 to 1.9	0.98*

°Based upon one way ANOVA analysis; *Based upon Chi-square test; SD, standard deviation; FVC, forced vital capacity; LLN, lower limit of normal; FEV_1, forced expiratory volume 1 second; n.a., non-applicable. Statistically significant p-values after application of Benjamini Hochberg correction (FDR 0,05) for multiple testing are highlighted in bold.

Suppl. Table 5 – Cross-sectional comparison of anti-SARS-CoV-2 antibodies between SARS-CoV-2 positive PIFS groups.

	unvaccinated PIFS+	***Vs.***	***unvaccinated PIFS-***	***p-value***
BAU/mL – median (IQR)	94.(1004)		146 (1269)	0.73
Nucleocapsid Antibody – median (IQR)	10 (6.8)		15 (29)	0.09
	vaccinated PIFS+	***Vs.***	***vaccinated PIFS-***	***p-value***
BAU/mL – median (IQR)	8219 (15223)		7864 (12852)	0.83
Nucleocapsid Antibody – median (IQR)	6.6 (7.5)		6.6 (11)	0.88

Based upon Mann Whitney; BAU, Binding Antibody Units; IQR, interquartile range

Suppl. Table 6 - Correlation between immunological markers and symptoms within the COVID+ PIFS+ group

		COVID-19 associated symptoms				
		Airway symptom score[a]	*Infection symptom score*[b]	*Cognitive symptom score*[c]	*Post-exertional malaise score*[d]	*Fatigue score*[e]
MCP-1	Corr. coef. (rho)	0.0101	-0.0042	0.0923	0.2125	0.0641
	p-value	0.9427	0.9761	0.5109	0.1267	0.6483
Eotaxin	Corr. coef. (rho)	0.0719	0.0971	0.1650	0.1831	0.1718
	p-value	0.6090	0.4891	0.2376	0.1895	0.2188

Spearman's rho correlation analysis with significant p-values after Benjamini-Hochberg correction (FDR 0,05) for test multiplicity highlighted in bold. [a] Sum of scores across the items “breathlessness”, “cough” and “runny nose”. [b] Sum of scores across the items “fever/chills”, “sore throat”, “headache”, “muscle ache” and “fatigue after exercise”. [c] Sum of scores across the items “decision-making”, “memory problems”, “concentration difficulty” and “confusion/disorientation”. [d] Sum of the DePaul Symptom Questionnaire items relating to post-exertional malaise. [e] Numerical sum of scores on the Chalder Fatigue questionnaire.

Suppl. Table 7 - Correlation between immunological markers and symptoms within the PCC+ group

		COVID-19 associated symptoms				
		Airway symptom score[a]	*Infection symptom score[b]*	*Cognitive symptom score[c]*	*Post-exertional malaise score[d]*	*Fatigue score[e]*
MCP-1	Corr. coef. (rho)	0.0237	0.0353	0.0302	0.1361	0.1063
	p-value	0.7275	0.6043	0.6574	0.0447	0.1185
Eotaxin	Corr. coef. (rho)	0.0596	0.1258	0.1141	0.1452	0.1201
	p-value	0.3812	0.0638	0.0930	0.0322	0.0776

Spearman's rho correlation analysis with significant p-values after Benjamini-Hochberg correction (FDR 0,05) for test multiplicity highlighted in bold. [a] Sum of scores across the items “breathlessness”, “cough” and “runny nose”. [b] Sum of scores across the items “fever/chills”, “sore throat”, “headache”, “muscle ache” and “fatigue after exercise”. [c] Sum of scores across the items “decision-making”, “memory problems”, “concentration difficulty” and “confusion/disorientation”. [d] Sum of the DePaul Symptom Questionnaire items relating to post-exertional malaise. [e] Numerical sum of scores on the Chalder Fatigue questionnaire.

Suppl. Table 8 – Cross-sectional comparison of white blood cell counts, cytokines and complement activation products between COVID-19 groups.

	LDL (pg/mL)	*COVID+*	*COVID-*	*p-value*
Plasma TNF, pg/mL – median (IQR)<br>C.I.	3.0	9.4 (14)<br>6.7 to 9.4	5.6 (14)<br>0.46 to 8.2	0.03**
Plasma MCP-1, pg/mL – median (IQR)<br>C.I.	0.40	5.2 (3.6)<br>4,6 to 5,6	2.1 (4.4)<br>1.7 to 3.3	**<0.0001****
Plasma IP-10, pg/mL – median (IQR)<br>C.I.	1.0	106 (65)<br>101 to 110	91 (59)<br>82 to 103	**0.0189****
Plasma Eotaxin, pg/mL – mean (SD)<br>C.I.	0.30	15 (6.6)<br>14 to 16	12 (5.9)<br>10 to 13	**<0.0001*****
Plasma MIP-1β, pg/mL – median (IQR)<br>C.I.	0.30	27 (37)<br>25 to 31	24 (27)<br>16 to 28	0.05**
Plasma RANTES, pg/mL – median (IQR)<br>C.I.	3.0	140 (169)<br>130 to 152	120,8 (141,5)<br>95 to 143	0.06**
Plasma IL-9, pg/mL – median (IQR)<br>C.I.	2.0	97 (150)<br>63 to 96	75 (121)<br>85 to 108	0.06**
Plasma GDF-15, ng/mL – mean (SD)<br>C.I.		0.45 (0.12)<br>0.43 to 0.46	0,.4 (0.12)<br>0.41 to 0.46	0.49***
Plasma hs-CRP, µg/mL – median (IQR)<br>C.I.		1.2 (3.8)<br>1.0 to 1.6	2,1 (5.0)<br>1.0 to 3.3	0.14**
Plasma TCC, CAU/mL – median (IQR)<br>C.I.		0.19 (0.13)<br>0.18 to 0.20	0.19 (0.17)<br>0.15 to 0.23	0.85**
Plasma C3bc, ng/mL – mean (SD)<br>C.I.		3.9 (1.5)<br>3.7 to 4.0	3,6 (1.5)<br>3.2 to 3.9	0.08***
Blood Platelet count, 10^9 cells/L - mean (SD)<br>C.I.		267 (59)<br>264 to 276	276 (58)<br>263 to 289	0.39***
Blood Leukocyte count, 10^9 cells/L - mean (SD)<br>C.I.		6.1 (1.7)<br>5.9 to 6.3	5.9 (1.5)<br>5.5 to 6.2	0.26***
Blood Lymphocyte count, 10^9 cells/L - mean (SD)<br>C.I.		2.0 (0.54)<br>2.0 to 2.1	2.0 (0.44)<br>1.9 to 2.1	0.58***
Blood Monocyte count, 10^9 cells/L - mean (SD)<br>C.I.		0.48 (0.15)<br>0.46 to 0.49	0.46 (0.13)<br>0.43 to 0.49	0.36***
Blood Neutrophil count, 10^9 cells/L - mean (SD)<br>C.I.		3.43 (1.5)<br>3.28 to 3.59	3.3 (1.3)<br>3.0 to 3.5	0.31***
Blood Eosinophil count, 10^9 cells/L – mean (SD)<br>C.I.		0.19 (0.18)<br>0.17 to 0.21	0.19 (0.16)<br>0.16 to 0.23	0.86***
Neutrophil-to-Lymphocyte ratio – mean (SD)<br>C.I.		1.82(0.98)<br>1.7 to 1.9	1.7 (0.69)<br>1.6 to 1.9	0.35***
SII - median (IQR)<br>C.I.		413 (284)<br>392 to 440	412 (317)<br>365 to 487	0.97**

• Based upon Chi Square test; ** Based upon Mann Whitney U; *** based upon Student T test; LDL, lower detection limit for multiplex assay; IQR, interquartile range; SD, standard deviation; GDF, growth/ differentiation factor; IL, interleukin; TNF, tumor necrosis factor; MCP, monocyte chemotactic protein; IP, interferon gamma-induced protein; MIP, macrophage inflammatory protein; hsCRP, high-sensitive assay of C-reactive protein; RANTES, Regulated on activation, normal T-cell expressed and secreted; C3b, complement component 3, part bc; TCC, terminal complement complex; CAU, complement activation unit; SII, systemic inflammatory index = Neutrophils x Platelets/Lymphocytes; Statistically significant p-values after application of Benjamini Hochberg correction (FDR 0,05) for multiple testing are highlighted in bold.

Suppl. Table 9 - Multiple linear regression modelling

		COVID-19 positive
Eotaxin	Coefficient	3.6
	(C.I. 95%)	(2.0 to 5.1)
	p-value	<0.001
	R- squared	0.13
MCP-1	Coefficient	2.4
	(C.I. 95%)	(1.6 to 3.1)
	p-value	<0.001
	R-squared	0.10
IP-10 - ln transformed	Coefficient	0.4
	(C.I. 95%)	(0.20 to 0.56)
	p-value	<0.001
	R-squared	0.03

Multiple linear regression focusing on eotaxin, MCP-1, IP-10 and COVID-19 status; All p-values remain significant after adjustment for possible confounders including age, sex, BMI and vaccination status; IL, interleukin; IP, interferon gamma induced protein; MCP, monocyte chemotactic protein; BMI, body mass index; C.I., confidence interval; BMI, body mass index.

Suppl. Table 10 – Cross-sectional comparison of spirometry results between SARS-CoV-2 groups.

	COVID+	***COVID-***	***p-value***
Number of samples	276	63	
FVC, % of predicted - mean (SD)<br>C.I	100 (12)<br>99 to 102	102 (9.4)<br>100 to 105	0.20*
FVC < LLN - no. (%)	15 (5.5)	1 (1.7)	0.21**
FEV_1, % of predicted - mean (SD)<br>C.I.	98 (12)<br>97 to 99	100 (8.5)<br>97 to 102	0.32*
FEV_1-to-FVC ratio - mean (SD)<br>C.I.	0.85 (0.07)<br>0.84 to 0.86	0.85 (0.05)<br>0.83 to 0.86	0.85*
FEV_1-to-FVC ratio < 0.7 - no. (%)	6.0 (2.2)	1.0 (1.7)	0.80**

*Based upon Student T test; **Based upon Chi square test; SD, standard deviation; FVC, forced vital capacity; LLN, lower limit of normal; FEV_1, forced expiratory volume 1 second.

Suppl. Table 11 - Correlation between immunological markers and symptoms within the COVID-19 positive group

		COVID-19 associated symptoms				
		Airway symptom score[a]	*Infection symptom score*[b]	*Cognitive symptom Score*[c]	*Post-exertional malaise score*[d]	*Fatigue score*[e]
MCP-1	Corr. coef. (rho)	-0.0163	-0.0685	-0.0764	0.0029	-0.0108
	p-value	0.7567	0.1909	0.1447	0.9561	0.8375
IP-10	Corr. coef. (rho)	0.0216	-0.0100	-0.0024	0.0226	0.0492
	p-value	0.6794	0.8480	0.9639	0.6667	0.3470
Eotaxin	Corr. coef. (rho)	-0.0315	-0.0034	-0.0677	-0.0569	0.0124
	p-value	0.5472	0.9489	0.1960	0.2791	0.8125

Spearman's rho correlation analysis with significant p-values after Benjamini Hochberg correction (FDR 0,05) for test multiplicity highlighted in bold. [a] Sum of scores across the items “breathlessness”, “cough” and “runny nose”. [b] Sum of scores across the items “fever/chills”, “sore throat”, “headache”, “muscle ache” and “fatigue after exercise”. [c] Sum of scores across the items “decision-making”, “memory problems”, “concentration difficulty” and “confusion/disorientation”. [d] Sum of the DePaul Symptom Questionnaire items relating to post-exertional malaise. [e] Numerical sum of scores on the Chalder Fatigue questionnaire.

Supplementary Figures

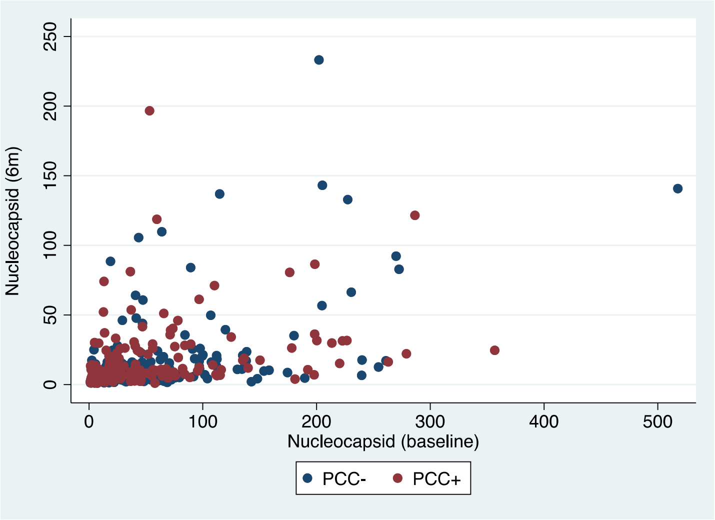

Suppl. Figure 1. Evolution of nucleocapsid titer in COVID-19 positive according to PCC caseness.
